# Targeting MicroRNA-143 Leads to Inhibition of Glioblastoma Tumor Progression

**DOI:** 10.3390/cancers10100382

**Published:** 2018-10-12

**Authors:** Eunice L. Lozada-Delgado, Nilmary Grafals-Ruiz, Miguel A. Miranda-Román, Yasmarie Santana-Rivera, Fatma Valiyeva, Mónica Rivera-Díaz, María J. Marcos-Martínez, Pablo E. Vivas-Mejía

**Affiliations:** 1 Department of Biology, Rio Piedras Campus, University of Puerto Rico, San Juan, PR 00931, USA; eunice.lozada@upr.edu (E.L.L.-D.); mirandar.miguel@gmail.com (M.A.M.-R.); yasmarie.santana@upr.edu (Y.S.-R.); 2 Department of Biochemistry, Medical Sciences Campus, University of Puerto Rico, San Juan, PR 00936, USA; mrivera@bromediconllc.com; 3 Comprehensive Cancer Center, University of Puerto Rico, San Juan, PR 00935, USA; nilmary.grafals1@upr.edu (N.G.-R.); fvaliyeva@cccupr.org (F.V.); 4 Department of Physiology, Medical Sciences Campus, University of Puerto Rico, San Juan, PR 00936, USA; 5 Department of Pathology and Laboratory Medicine, Medical Sciences Campus, University of Puerto Rico, San Juan, PR 00936, USA; maria.marcos@upr.edu; 6 Anatomic Pathology Laboratory, Puerto Rico Medical Services Administration, San Juan, PR 00936, USA

**Keywords:** glioblastoma, microRNAs, mouse model, cell proliferation

## Abstract

Glioblastoma (GBM) is the most common and aggressive of all brain tumors, with a median survival of only 14 months after initial diagnosis. Novel therapeutic approaches are an unmet need for GBM treatment. MicroRNAs (miRNAs) are a class of small non-coding RNAs that regulate gene expression at the post-transcriptional level. Several dysregulated miRNAs have been identified in all cancer types including GBM. In this study, we aimed to uncover the role of miR-143 in GBM cell lines, patient samples, and mouse models. Quantitative real-time RT-PCR of RNA extracted from formalin-fixed paraffin-embedded (FFPE) samples showed that the relative expression of miR-143 was higher in GBM patients compared to control individuals. Transient transfection of GBM cells with a miR-143 oligonucleotide inhibitor (miR-143-inh) resulted in reduced cell proliferation, increased apoptosis, and cell cycle arrest. SLC30A8, a glucose metabolism-related protein, was identified as a direct target of miR-143 in GBM cells. Moreover, multiple injections of GBM tumor-bearing mice with a miR-143-inh-liposomal formulation significantly reduced tumor growth compared to control mice. The reduced in vitro cell growth and in vivo tumor growth following miRNA-143 inhibition suggests that miR-143 is a potential therapeutic target for GBM therapy.

## 1. Introduction

Glioblastoma (GBM), also known as glioblastoma multiforme, is the most common and lethal form of brain tumor [1]. Currently, there are no optimal treatments for this disease, which accounts for about 14,000 annual deaths in the U.S., having an incidence ratio of 2 to 3 out of 100,000 adults per year [2]. GBM, or WHO Grade IV astrocytoma, can either develop de novo (primary, 90% of cases) or derive from WHO grade II or grade III astrocytomas (secondary) [1,3,4,5]. GBM tumors are known to be fast growing in the cerebral white matter and patients typically remain asymptomatic until advanced stages of the disease [6]. The current therapeutic strategy for GBM involves tumor resection surgery followed by radiotherapy (XRT) and/or radiosurgery in combination with temozolomide (TMZ)-based chemotherapy [1,7]. However, most GBM patients become resistant to a second round of TMZ treatment due to over-activation of the DNA repair enzyme O6-methylguanine-DNA methyltransferase (MGMT) [2,8]. Despite the aggressive treatment, the prognosis of GBM patients remains poor, with survival rates of only 12–14 months after initial diagnosis. Therefore, novel and more effective therapies for GBM are urgently needed.

MicroRNAs (miRNAs) are small non-coding RNAs of about 18–22 nucleotides in length that regulate gene expression post-transcriptionally by recognizing and binding preferably to the 3’-Untranslated Region (3’-UTR) of their target messenger RNAs (mRNAs) [9]. In addition, miRNAs binding to the 5’-UTR or coding regions have also been observed [9,10,11]. Evidence indicates that altered expression of miRNAs in GBM plays a central role in GBM initiation, progression, and tumor maintenance [12,13,14]. This has led to the proposal of several miRNAs as both diagnostic and prognostic markers, and as targets for GBM therapy [2].

MicroRNA-143 (miR-143) is part of a conserved miRNA cluster composed of miR-143/miR-145 on chromosome 5 (5q33) in humans [15]. MiR-143 has been shown to have a role in tumor progression, cancer cell growth, and invasiveness of cancer cells, including GBM cells [16,17]. As most miRNAs, miR-143 expression appears to be tissue-specific [17,18]. In normal tissues, miR-143 expression ranges from highest in the colon to lowest in the brain and liver [15]. Reports have shown that high levels of miR-143 sensitize cells to chemotherapeutic drugs including docetaxel in prostate cancer cells and TMZ in GBM cells [8,19]. However, others show an association between high levels of miR-143 with increased invasive potential of GBM cells compared with parental GBM cells, suggesting an oncogenic role [16]. Since the biological role of miR-143 in GBM is not well understood, in the present study we rigorously investigated the role of miR-143 in GBM cell lines, patient samples, and a subcutaneous GBM mouse model.

## 2. Results

### 2.1. Expression of MiR-143 in GBM Patients

**Figure 1:**
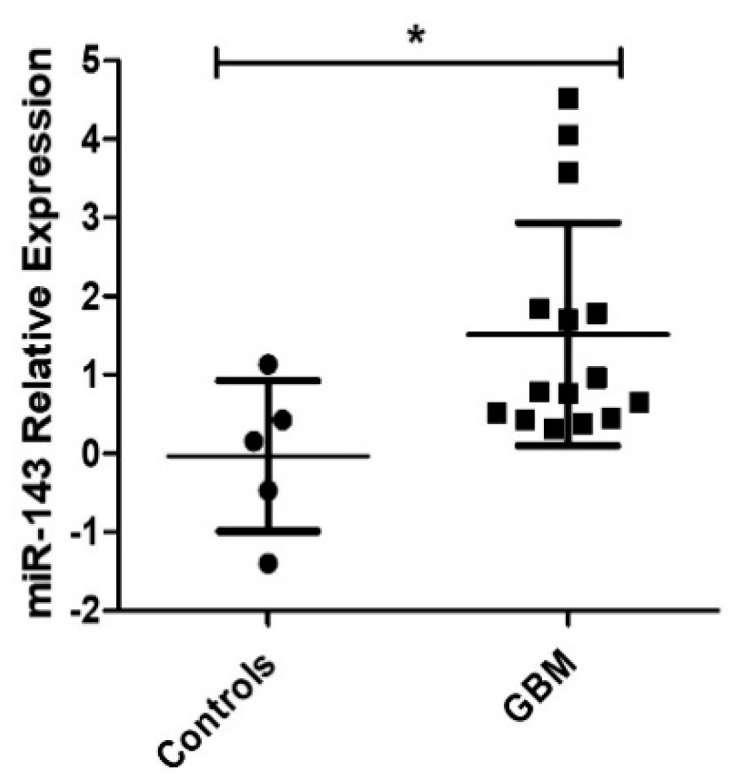
MiR-143 expression levels in GBM. Formalin-fixed paraffin-embedded (FFPE) tissue blocks from 19 newly diagnosed Glioblastoma (GBM) patients (13 females, 6 males) and 5 control patients (2 females, 3 males) were used in this study. GBM patients showed higher miR-143 expression compared to control patient samples (* *p* < 0.05); dots represent the means of triplicates ± SD.

First, we assessed the miR-143 expression levels in FFPE samples of GBM patients [14,20,21]. MiR-143 expression levels were found to be significantly increased in GBM patients compared to controls (* *p* = 0.0208) (Figure 1).

### 2.2. Effect of MiR-143 Targeting on GBM Cell Proliferation

**Figure 2:**
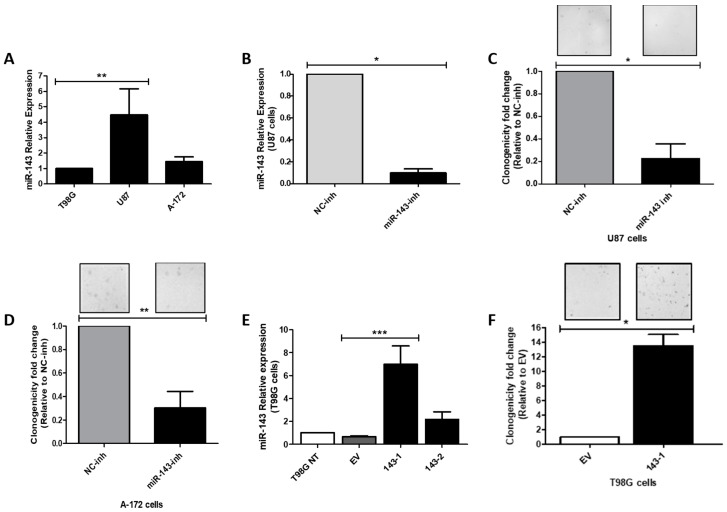
Effect of miR-143 inhibition or overexpression on cell proliferation. Total RNA was isolated, and qPCR was performed. (**A**) Relative miR-143 expression in a panel of Glioblastoma (GBM) cell lines, calculated relative to T98G cells; (**B**) Relative miR-143 expression after transient transfection of U87 with miR-inhibitors, calculated relative to the Negative control (NC) inhibitor. Colony formation assay after transfection of (**C**) U87 and (**D**) A-172 GBM cells with miR-143 inhibitor (miR-143-inh) or negative control inhibitor (NC-inh). (**E**) qPCR for relative miR-143 expression in empty vector (EV) and miR-143 T98G clones, calculated relative to the T98G non-treated (T98G NT) cells. (**F**) Colony formation assay of the T98G (143-1) miR-143 overexpressing clone and T98G Empty Vector (EV) clone. Columns represent the means of at least triplicates ± SEM (* *p* < 0.05, ** *p* < 0.01, *** *p* < 0.001).

We measured miR-143 expression levels in a panel of three well-known GBM cell lines (U87-MG, T98G, A-172). MiR-143 was found to be expressed in higher levels in the U87-MG (U87) cell line, while the T98G cells expressed the lowest miR-143 levels (Figure 2A). Therefore, the U87 cell line was used for miR-143 inhibition experiments, while the T98G cell line was chosen for ectopic miR-143 overexpression. To examine the effect of targeting miR-143 on cell proliferation, U87 cells were transiently transfected with 100 nM of oligonucleotide-inhibitors (miR-143-inh or NC-inh). Following this treatment, miR-143 levels were significantly reduced in U87 cells by ~90% (* *p* = 0.0211; Figure 2B). These cells showed a reduced ability to proliferate as shown by the 78% reduction in the number of colonies (* *p* = 0.0202) compared to the NC-inh transfected cells (Figure 2C). Transient transfection of A-172 cells with 100 nM of miR-143-inh produced similar cell growth inhibitory effects (Figure 2D).

Taq-Man-based RT-PCR (qPCR) analysis with the total RNA extracted from stable transfected clones showed a 6.98-fold and 2.17-fold increase (compared with empty vector clones) in two of the miR-143 selected clones (Figure 2E). In a colony formation assay, the miR-143-overexpressed clone miR-143-1 grew significantly faster than cells expressing the empty vector clones (EV) (Figure 2F). Together, these results suggest that high miR-143 levels promote cell proliferation of GBM cells.

### 2.3. Effect of MiR-143 Targeting on Cell Cycle Progression and Apoptosis

**Figure 3:**
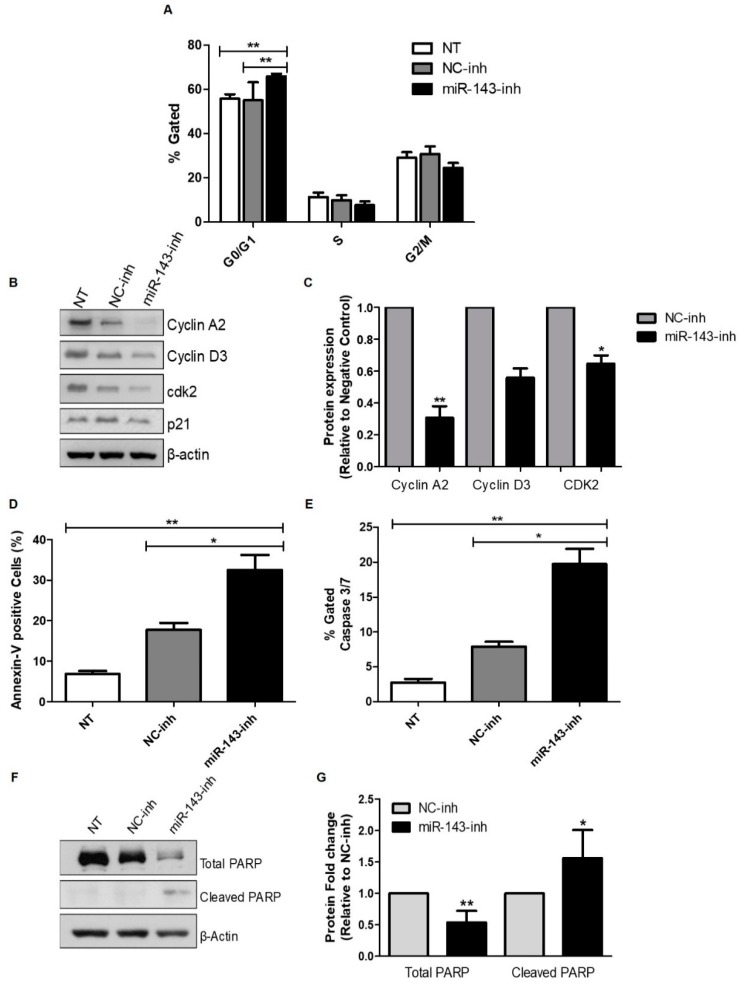
Inhibition of miR-143 induces apoptosis and cell cycle arrest. Apoptosis and cell cycle progression were measured by flow cytometry as described in the “Materials and Methods” section. U87 cells were transfected with 100 nM of negative control (NC-inh) or miR-143 inhibitor (miR-143-inh). (**A**) Seventy-two hours later, cells were fixed and cell cycle progression was assessed using the Muse Cell Analyzer. (**B**) Western blot analysis was performed 72 h after miR-inh transfection to detect changes in cell cycle-related proteins. (**C**) Densitometric analysis of the band intensities from (**B**) was performed and intensity values were expressed relative to NC-inh treated cells. U87 cells were treated as in (**A**), and 72 h later the Muse Cell Analyzer was used to measure apoptosis with (**D**) Annexin V and (**E**) Caspase 3/7 activity assays. (**F**) U87 cells were treated as in (**A**) and western blot analysis was performed to detect PARP-1 expression. (**G**) Densitometric analysis of the band intensities from (**F**) was performed and values were expressed relative to NC-inh treated cells. Columns represent the means of at least triplicates ± SEM (* *p* < 0.05, ** *p* < 0.01).

Next, we investigated whether the reduction of cell proliferation after miR-143 downregulation was due to activation of apoptosis and/or inhibition of cell cycle progression. Transient transfection of miR-143-inh in U87 GBM cells produced a cell cycle arrest in the G0/G1 to S phase 72 h post-transfection (Figure 3A). These results were further validated by western blot analysis of key proteins involved in G0/G1 to S phase transition. Particularly, cyclin A2, cyclin D3, and cyclin-dependent kinase 2 (Cdk2) protein levels decreased following miR-143 inhibition (Figure 3B,C). 

Furthermore, compared to NC-inh transfected cells, treatment of U87 cells with the miR-143-inh significantly increased apoptosis as evidenced by increased Annexin-V positive cells (Figure 3D) and caspase 3/7 levels (Figure 3E). Western blot analysis confirmed the induction of apoptosis showing a drastic reduction in the total poly (ADP-ribose) polymerase (PARP) protein levels accompanied by a significant increase in cleaved PARP (Figure 3F,G). Together, these results suggest that high levels of miR-143 protect GBM cells from apoptotic cell death.

### 2.4. MiR-143 Target Prediction and Validation

**Figure 4:**
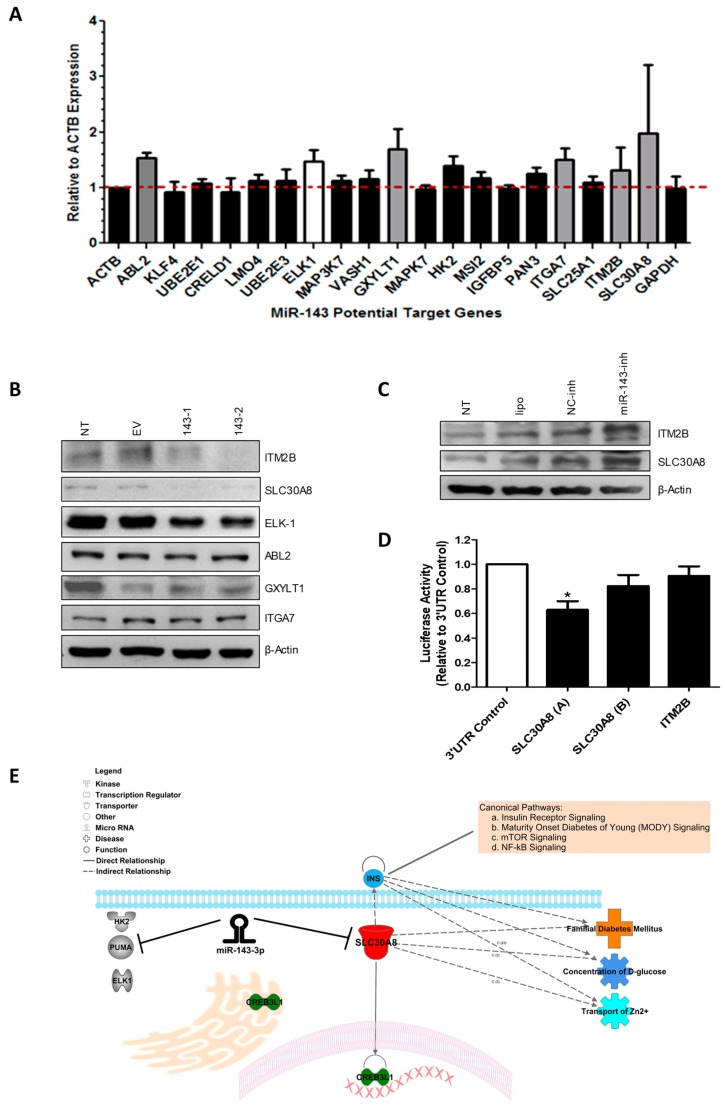
Identification of miR-143 target genes in GBM cells. (**A**) SYBR Green-based qPCR was performed with total RNA isolated from T98G cells transiently transfected with miR-143-inh or NC-inh. (**B**) Western blot analysis was performed with protein extracts from miR-143 overexpressed (143-1, 143-2) and EV clones. (**C**) Western blot analysis of protein extracts from U87 cells treated with 200 nM miR-143-inh or NC-inh, Non-treated cells (NT), and U87 cells with transfection reagent alone (lipo) as loading controls. (**D**) Dual-luciferase reporter assays were performed where luciferase activity was calculated relative to the NC-inh. (**E**) IPA analysis showing the interaction of miR-143 with its target genes and SLC30A8 association with glucose metabolism.

To identify further miR-143 target mRNAs in GBM cells, we performed in silico analysis using six different microRNA target identification softwares (Targetscan, Diana microT, miRPath, miRecords, miRDIP, miRGator). Over a hundred potential miR-143-regulated genes were identified by this approach (data not shown). Targets identified by at least three softwares generated a list of 21 potential miR-143-regulated genes (Figure 4A). Total RNA extracted from T98G cells transiently transfected with 100 nM of miR-143-inh or NC-inh was used for SYBR Green I-based qPCR analyses. Compared with the NC-inh transfected cells, transient transfection of miR-143-inh increased the mRNA levels of 6 out of the 21 miR-143 potential target genes (Figure 4A). These genes include SLC30A8, ITGA7, GXYLT1, ABL2, and ITM2B (Table 1). ELK-1 has been previously reported as a direct miR-143-regulated gene and was used as a positive control [22]. Western blot analysis was performed with protein lysates from miR-143 overexpressed clones 143-1 and 143-2 (Figure 2E). A reduction in the protein levels of ITM2B, SLC30A8, and ELK-1 was observed in the miR-143 overexpressed clones (143-1, 143-2) compared to EV or non-treated (NT) protein samples (Figure 4B). However, no observable changes in protein levels were detected for ABL2, GXYLT1, or ITGA7 in the samples tested (Figure 4B). Moreover, western blot analysis of U87 cells transiently transfected with 200 nM of miR-143-inh showed increases in expression of both ITM2B and SLC30A8 compared to the NC-inh U87 transfected cells (Figure 4C). These results suggest that both ITM2B and SLC30A8 are regulated by miR-143, either directly or indirectly.

Dual-luciferase reporter assays were performed to confirm that miR-143 binds directly to the 3′-UTR of the mRNA of ITM2B and SLC30A8. A plasmid containing the 3′-UTR region of each mRNA (ITM2B or SLC30A8) and a plasmid containing the pre-miR-143 were co-transfected, as described in the “Materials and Methods” section. The 3′-UTR of SLC30A8 was divided into two vectors due to its length (A and B). The sequence of the A and B 3′-UTR fragments are shown in Supplementary Figure S1. A reduction of 37% in the relative luciferase activity of the SLC30A8 (A) 3′-UTR (* *p* < 0.05) was observed, compared to the 3′-UTR control vector confirming direct binding of miR-143 to this target mRNA (Figure 4D). Supplementary Figure S2 shows the binding region of miR-143 to SLC30A8. However, changes in luciferase activity for ITM2B were not observed (Figure 4D). Ingenuity pathway analysis (IPA) shows that SLC30A8 is a zinc transporter protein associated with glucose metabolism (Figure 4E) [23]. Moreover, SLC30A8 has been shown to interact with CREB3L1 (cAMP responsive element binding protein 3-like 1), a transcription factor related to unfolded protein response of the Golgi apparatus and endoplasmic reticulum stress [24].

### 2.5. In Vivo Targeting of MiR-143

**Figure 5:**
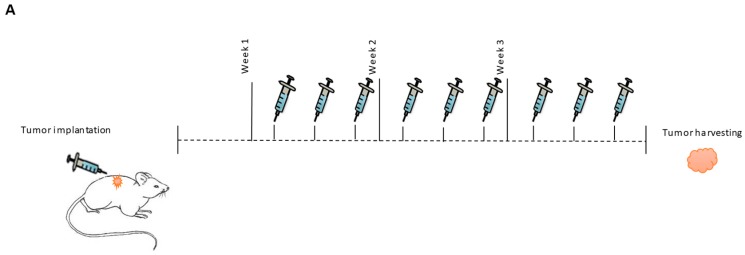

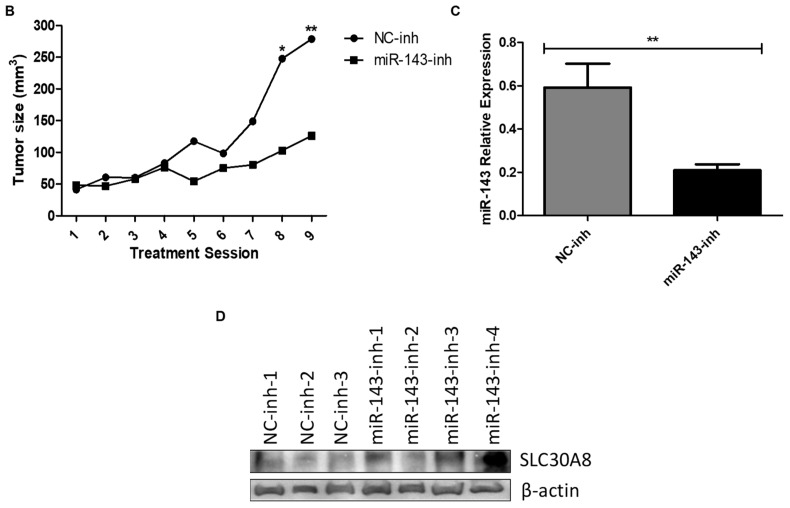
In vivo targeting of miR-143 reduces tumor growth. (**A**) Experimental design. (**B**) MiR-143 inhibition effect on tumor growth was calculated as described in the “Materials and Methods” section. (**C**) MiR-143 expression levels were measured by qPCR from total RNA extracted from mouse tumor tissues. Columns represent the means of N = 7 for NC-inh and N = 9 for miR-143-inh treatments ± SEM. (**D**) SLC30A8 expression levels were measured by western blot analysis in protein samples extracted from the mouse tumor tissues. * *p* < 0.05, ** *p* < 0.01.

We then assessed the effect of targeting miR-143 on tumor progression in a GBM subcutaneous mouse model. MiRNA inhibitors were administered as a DOPC-PEG-cholesterol-based nanoliposome formulation [37]. The experimental design is illustrated in Figure 5A. Tumor-bearing mice were divided into two groups: DOPC-PEG-liposomal-miR-NC-Inh (N = 7) and DOPC-PEG-liposomal-miR-143-Inh (N = 9). Nine days after cell implantation, nude mice bearing U87 tumors were injected *i.p.* three times a week for three weeks. Each dose consisted of 2.5 µg of DOPC-PEG-liposomal-miR-NC-Inh or 2.5 μg of DOPC-PEG-liposomal-miR-143-Inh. Liposomal miR-143-inh treatment reduced tumor growth compared to the control group (Figure 5B). The differences among groups were statistically significant, particularly for the last two treatment sessions. To confirm that the reduction in tumor growth was caused by the inhibition of miR-143, we assessed the miR-143 expression levels in total RNA extracted from mouse tumor samples of both NC-inh and miR-143-inh treated groups. qPCR results showed a significant reduction in miR-143 expression levels in the miR-143-inh group compared to the NC-inh group (Figure 5C). Western blot analysis of protein extracted from mouse tumor samples showed increased SLC30A8 protein levels in three out of the four miR-143-inh samples (miR-143-inh-1, 3, 4) compared to the NC-inh group (Figure 5D). These results further confirm that SLC30A8 is a miR-143 target in GBM cells.

## 3. Discussion

The major findings of this study are that miR-143 levels are increased in GBM patient samples compared to normal controls; and that targeting miR-143 reduced cell growth in vitro and tumor growth in a xenograft mouse model of GBM. Early studies showed divergences regarding the role of miR-143 in cancer [38]. In colorectal cancer, low levels of miR-143 have been associated with exacerbation of cancer proliferation in patient samples and cell lines [39]. Similar results were obtained in gastric cancer [40]. In contrast, a recent study showed that miR-143 expression in stromal cells of a mouse model of lung adenocarcinoma promoted tumor progression through neoangiogenesis [38]. Similarly, Koo et al. showed that serial selection for invasiveness in glioblastoma cell lines (U87, U251, U373, and rat glioma cell line C6) exhibited increased expression of miR-143 compared with parental GBM cells, suggesting an invasive oncogenic role of miR-143 in these cells [16].

While Wang and colleagues found that miR-143 is down-regulated in GBM tissues compared to normal brains [8], we obtained opposite results. Differences in the integrity of the tissue samples, the internal standard used for the real-time PCR experiments, and miRNA polymorphisms could contribute to these divergent results [41,42,43]. Furthermore, the presence of multiple cancer cell populations in a tumor (tumor heterogeneity) may result in diverse miRNA expression patterns, as evidenced by other miRNA expression profiles [2,44].

We observed a reduction in cell proliferation, activation of apoptosis, and cell cycle arrest following miR-143 inhibition. These results support the conclusion that high levels of miR-143 promote cell proliferation and protect GBM cells from apoptosis. In fact, PUMA (p53 upregulated modulator of apoptosis), a reported miR-143-target, is a proapoptotic protein that has been demonstrated to reduce the growth of subcutaneous U87 tumors in nude mice [45,46,47]. In our study, multiple injections of a liposomal-miR-143-Inh reduced tumor growth in a subcutaneous GBM mouse model. A similar cancer-driving role for miR-143 is supported by other studies, including Koo et al., where up-regulation of miR-143 increased the invasive ability of GBM cells [16,38]. These results corroborate the oncogenic role of miR-143 in GBM cells.

In this study, we identified SLC30A8 as a direct target of miR-143 in GBM cells. SLC30A8, or Solute carrier family 30 (zinc transporter) member 8, is a protein highly expressed in pancreatic beta cells. Evidence indicates that SLC30A8 is necessary for the transport of cytosolic zinc into insulin granules for further insulin maturation (Figure 5E) [25,48]. Interestingly, miR-143 has been reported to promote type 2 diabetes and adipocyte differentiation [49]. In different tumor types including breast, colon, and in GBM stem-like cells, miR-143 has been linked to glucose metabolism and regulation of cancer glycolysis via targeting hexokinase-2 [50,51,52,53,54]. As metabolic rewiring has been recently coined as a hallmark of cancer [55], further studies should be performed to uncover the role of miR-143 and its target gene SLC30A8, in metabolism pathways contributing to GBM progression and tumor maintenance. Again, as the expression of miRNA-143 (as most miRNAs) is tissue- and time-dependent, its role in the intracellular glucose accumulation and the progression of GBM should be carefully interpreted [2]. Of note, recent reports indicate that the circular RNA, DLGAP4 (circDLGAP4) [56]; and the Mir-143 host gene (MIR143HG) [57] act as miRNA sponges that sequester miRNA-143, thus, reducing the ability of this miRNA to bind to its cognate targets.

Moreover, miR-143 post-transcriptionally regulates other genes including HK2, PUMA, and ELK-1 [22,46,50,51,52,53]. The decreased expression of all these genes (including SLC30A8), as a result of the aberrant overexpression of miR-143, could contribute to the uncontrolled growth, proliferation, and tumor maintenance of GBM cells. Although direct binding of miR-143 to the mRNA of ITM2B was not observed, the changes in ITM2B protein levels upon miR-143 manipulation suggest that this protein is a miR-143 downstream effector in GBM cells. ITM2B is known as a tumor suppressor that triggers p53-independent apoptosis [33,36]. The molecular mechanism of how ITM2B is regulated by miR-143 in GBM cells should be further investigated.

## 4. Materials and Methods

### 4.1. Tumor Samples: RNA and Protein Isolation

Formalin-fixed paraffin-embedded (FFPE) tissue blocks (2008–2010) from 19 newly diagnosed (de novo) glioblastoma patients (12 females, seven males; median age 59) and five control patients (two females, three males; median age 38) were obtained from the Pathology Department of the University of Puerto Rico-Medical Sciences Campus. The control samples were from patients selected based on having non-neoplastic, non-infectious brain tissue with no metabolic conditions and without hemorrhagic or necrotic diathesis. The research protocol conducted was approved by the University of Puerto Rico-Medical Sciences Campus Institutional Research Board (IRB) on 26 January 2016 (protocol number: A9180112). A representative hematoxylin and eosin (H&E)-stained slide from each of the selected tissue blocks was evaluated by a pathologist and neurooncologist to corroborate diagnosis and delineate tumor tissue from normal brain tissue. RNA isolation followed the protocol previously described by Rivera et al. [14]. Briefly, for each FFPE tissue block, a 3-mm punch biopsy sample was obtained from the tumor area previously delineated by the pathologist. Samples were processed for total RNA isolation using the RecoverAll Total Nucleic Acid Isolation Kit (Ambion, Austin, TX, USA). RNA concentrations were measured using NanoDrop (Thermo Scientific, Wilmington, DE, USA). The threshold cycles (Ct) were used to calculate the relative miR-143 expression using U48 as the internal control [14,58]. RNA was isolated from mice tumor tissue with the RecoverAll Total Nucleic Acid Isolation kit as per the manufacturer instructions. Protein extraction from mice tumor tissue was performed using the same procedure of protein isolation described below. In both cases, the frozen tumor tissue was placed in liquid nitrogen, followed by tissue homogenization with a mortar and electric tissue homogenizer (D1000 Hand-held homogenizer, Benchmark Scientific, Sayreville, NJ, USA).

### 4.2. Cells and Culture Conditions

T98G, U87-MG (U87), and A-172 GBM human cancer cells (RRID: CVCL_0556, CVCL_0022, and CVCL_0131, respectively) were purchased from American Type Culture Collection (ATCC, (Manassas, VA, USA). These lines were authenticated by the ATCC on August 2016. The cells were maintained in adherent culture in Dulbecco’s Modified Eagle Medium: Nutrient Mixture F-12 (DMEM/F-12; GIBCO) (Invitrogen Corporation) supplemented with 10% fetal bovine serum (FBS) (Thermo Scientific, Logan, UT, USA), 100 U/mL penicillin/streptomycin (Thermo Scientific) at 37 °C in a humidified chamber with 5% CO_2_. In vitro assays were performed at 75−85% cell density in passages 1−8.

### 4.3. Transient and Stable Transfections

For transient transfections, U87 cells (3 × 10^4^ cells/mL) were plated in 6-well plates or Petri dishes. After 24 h, a mixture of 100 nM (final concentration) of miRNA oligonucleotide-inhibitors (negative control and miR-143) (Life Technologies, Grand Island, NY, USA), lipofectamine RNAiMAX (Life Technologies) (1:1 ratio, v/v), and Opti-MEM I (Life Technologies) was added to the cells for 6−8 h. Then, the media was changed to regular DMEM with 10% FBS. For stable transfections, T98G cells were plated in a 6-well plate (3 × 10^4^ cells/mL). After 24 h, a mixture of 3 µg of miR-143 (pCMV-MIR143) or Empty (pCMV-EV) OriGene vectors (OriGene Technologies, Inc. Rockville, MD), lipofectamine RNAiMAX (Life Technologies) (1:1 ratio, v/v), and Opti-MEM I (Life Technologies) was added to the cells for 6−8 h. These vectors contain a Neomycin resistance cassette, which was used for mammalian cell clone selection and maintenance. Independent clones were picked and grown individually. RNA was isolated using the mirVana miRNA Isolation Kit (Ambion, Austin, TX, USA), following manufacturer’s instructions. To monitor miR-143 expression in empty vector and miR-143-overexpressing clones we used Taqman based qPCR (Applied Biosystems, Life Technologies, NY, USA). Threshold cycles (Ct) were used to calculate the relative miR-143 expression using U48 as an internal control [14,58].

### 4.4. Colony Formation Assay

Cell proliferation was assessed by a clonogenic assay. U87 cells were seeded in a 6-well plate (3.0 × 10^4^ cells/mL). The next day cells were transiently transfected with miR-143 oligonucleotide-inhibitor (miR-143-inh) or negative-control inhibitor (NC-inh) as described above. The next day cells were collected, and 1000 cells were seeded in 10 cm-Petri dishes. Ten days later, colonies were stained with 0.5% crystal violet in methanol and counted using an Eclipse TS100 microscope (Nikon, Minato, Tokyo, Japan) [59]. Colony formation assay was also performed with T98G miR-143-overexpressed clones without the oligonucleotide-inhibitor transfection step.

### 4.5. Assessment of Cell Cycle and Apoptosis

U87 cells were transiently transfected with a 100 nM (final concentration) of miR-143-inh or NC-inh as described above. Apoptosis was evaluated by flow cytometry analysis 72 h post-transfection using both Muse Annexin V Dead Cell Kit and Caspase 3/7 Kits (EMD Millipore Headquarters, Burlington, MA, USA). Cell cycle progression was evaluated by flow cytometry 72 h post-transfection with the Cell Cycle Kit (EMD Millipore Headquarters). Data was analyzed and collected using the Muse Cell Analyzer (EMD Millipore Headquarters). For western blot analysis, U87 transiently transfected (miR-143-inh or NC-inh oligonucleotides) cells were collected for western blot analysis.

### 4.6. MiRNA Target Prediction and SYBR-Green I RT-PCR for Target Identification

Online target prediction softwares (microrna.org, Diana-microT, Target Scan Human, mirPath, miRecords, miRGator, and mRDB) were used to identify potential target genes of miR-143. Total RNA from miR-143-inh and NC-inh samples were subjected to reverse transcription using the iScript cDNA Synthesis Kit from Bio-Rad Laboratories (Hercules, CA, USA). Data was collected and analyzed using a StepOne Software v2.1 from Applied Biosystems following manufacturer’s protocol (95 °C for 2 min followed by 40 cycles of 5 s at 95 °C and 30 s at 60 °C). β-actin was used as the internal standard for the gene expression values [59,60]. We used the multalin software (http://multalin.toulouse.inra.fr/ multalin/) [61] for alignment analysis of miR-143-3p (mature strand) and the SLC30A8(A) fragment mRNA.

### 4.7. Western Blot Analysis

Cells were collected, washed with PBS 1X, and stored at −80 °C until processed. For protein extraction, cells were lysed on ice with lysis buffer (1% Triton X, 150 mM NaCl, 25 mM Tris HCl, 0.4 mM NaVO_4_, 0.4 mM NaF and protease inhibitor cocktail from Sigma, St. Louis, MO, USA) for 30 min vortexing periodically. Lysates were centrifuged for 15 min at 4 °C, supernatants collected, and total protein concentration was determined using Bio-Rad DC Protein Assay reagents (Bio-Rad) following the manufacturer’s protocol. Equal protein quantities for each sample (30 or 50 μg per lane) were separated by SDS-PAGE, blotted onto nitrocellulose membranes, blocked with 5% non-fat milk, and probed with the appropriate dilution of the corresponding primary antibody. Once incubated with the primary antibody, membranes were rinsed and incubated with the corresponding HRP-conjugated secondary antibody. Bound antibodies were detected using an enhanced chemiluminescence substrate followed by autoradiography using a FluorChemTM 8900 (Alpha Innotech Corporation, San Leandro, CA, USA). Primary antibodies used: anti-ITM2B (1C11) (3 0kDa), anti-ELK-1 (E277) (45 kDa), anti-ITGA7 (129 kDa), anti-ABL2 (EPR1222(2)) (128 kDa), anti-SLC30A8 (40 kDa), anti-GXYLT1 (51 kDa) (Abcam, Cambridge, UK); cdk2 (78B2] (33 kDa), Cyclin D3 (DCS22) (31 kDa), p21 (12D1) (21 kDa), Cyclin A2 (BF683) (55 kDa), PARP (89, 116 kDa) (Cell Signaling, Danvers, MA, USA); and anti-β-actin (42 kDa) (Sigma). Secondary antibodies used: anti-mouse and anti-rabbit IgG horseradish peroxidase (HRP) (Cell Signaling).

### 4.8. Dual-Luciferase Reporter Assays

T98G cells (3.5 × 10^4^ cells/mL) were transiently transfected with 1.5 μg pre-miR vectors (miR-143 and empty vector control) and lipofectamine RNAiMax (1:1 ratio, v/v) in Opti-MEM I media. After 6 h of transfection cells were washed with PBS 1X and the second transfection of 1.5 μg of dual Firefly/Renilla luciferase reporter mammalian expression vectors (pEZX-MT06; GeneCopoeia, Rockville, MD, USA) was performed. Vectors included the 3’-UTR of SLC30A8 (in two portions-A and B), ITM2B, and an empty vector control. The transfection mix included the 3’-UTR vector, lipofectamine RNAiMax (Life Technologies) (1:1 ratio, v/v) and Opti-MEM I which was added for another 6 h. Then, the media was changed to fresh DME/F-12 (10% FBS and 0.1% penicillin/streptomycin). After 48 h firefly and renilla activity were measured in a Glomax 20/20 luminometer using the Dual-Luciferase Reporter Assay System kit (Promega, Madison, WI) following the manufacturer’s protocol. The relative luciferase activity was calculated and graphed relative to the negative-control inhibitor samples with each 3’-UTR vector.

### 4.9. Tumor Implantation and Treatment

Male (10) and female (10) BALB/c nude mice of 4 to 6 weeks of age were purchased from Taconic Biosciences (Rensselaer, NY, USA). U87 cells were subcutaneously (*s.c*.) injected into the right dorsal flank (2.0 × 10^6^ cells/200 μL in PBS/Matrigel mixture). Tumor size was measured each treatment session (every three days) with a caliper. Tumor volumes were calculated using the following formula: volume = (L × W × H) × 0.5, where L is the length (longest diameter), W is the weight (thickness), and H is the height (shorter diameter). Liposomal-miRNA-inh administration initiated once tumors were visible (after 7 days). Mice were injected intraperitoneally (*i.p*.) three times a week for three weeks with DOPC-PEG-liposomal-miR-NC-Inh or DOPC-PEG-liposomal-miR-143-Inh. Liposomes were composed of miRNA-inh; 1,2-dioleoyl-sn-glycero-3-phosphocholine (DOPC) (1:10 w/w ratio); (1,2-distearoyl-sn-glycero-3-phosphoethanolamine-*N*-(amino(polyethylene glycol)-2000) (ammonium salt) − (DSPE-PEG-2000) (5% mol/mol of DOPC); and cholesterol (25% w/w of DOPC). This liposome formulation has been previously described [37]. At the end of treatment, mice were euthanized, tumors were measured and weighted. Tumor samples were processed, and total RNA extraction was obtained with the mirVana miRNA Isolation Kit (Life Technologies, Thermo Fisher Scientific, Waltham, MA, USA) as per de protocol instructions. MiR-143 and U48 (internal control) were measured by real-time PCR with TaqMan specific probes (Applied Biosystems). Animal handling and research protocols were approved by the Institutional Animal Care and Use Committee (IACUC) of the University of Puerto Rico, Medical Sciences Campus on 10 April 2018 (protocol number: A870110).

### 4.10. Statistical Analysis

Statistical analysis was performed using GraphPad Prism 5 (GraphPad Software, Inc., La Jolla, CA, USA). Data was analyzed using Student’s t-test for comparing two groups and ANOVA tests for multiple group comparisons, with *p* < 0.05 considered statistically significant (* *p* < 0.05, ** *p* < 0.01, *** *p* < 0.001). All experiments were performed at least in triplicates.

## 5. Conclusions

We provide evidence that miR-143 levels are increased in GBM patients and that miR-143 acts as an oncogene by promoting cell proliferation and survival of GBM cells. Further preclinical studies using orthotopic xenograft models and direct administration of the miR-143 inhibitor into an orthotopic brain tumor should be conducted to confirm that miR-143 is a viable molecular target for GBM treatment. We also identified SLC30A8 as a direct target mRNA of miR-143 in GBM cells in vitro and in vivo. Further studies should elucidate the role of this novel cancer-associated gene in GBM.

## Figures and Tables

**Table 1:** List of the five miR-143 predicted target genes from qPCR results.

Full Name	Gene Symbol	Fold Change miR-143-Inh vs. NC-Inh in T98G Cells	Biological Role
Solute carrier family 30 (zinc transporter), member 8	SLC30A8	1.98	Role in glucose homeostasis; diabetes mellitus [25,26].
Integrin, alpha 7	ITGA7	1.51	Receptor for the basement membrane protein laminin-1 [27]; Tumor suppressor gene [28].
Glucoside xylosyltransferase 1	GXYLT1	1.69	Notch xylosyltransferase [29].
ABL proto-oncogene 2, non-receptor tyrosine kinase	ABL2	1.53	Oncogene related to cell migration and invasion in various cancers; role in cytoskeletal rearrangement, regulator of neuronal structural stability [30,31,32].
Integral membrane protein 2B	ITM2B	1.32	Role in triggering apoptosis P53 independent; tumor suppressor [33]. Transmembrane protein involved in negative regulation of amyloid processing. Related to dementia [34,35,36].

## Supplementary Materials

Supplementary materials can be found at http://www.mdpi.com/2072-6694/10/10/382/s1, Figure S1: SCL30A8 3′UTR vectors A and B sequences, Figure S2: Sequence alignment of miR-143 and SLC30A8 3′UTR.
