# Supplementary material for: Targeting MicroRNA-143 Leads to Inhibition of Glioblastoma Tumor Progression
Source: Cancers (Basel). 2018 Oct 12;10(10):382. doi: 10.3390/cancers10100382 (PMC6210372; doi:10.3390/cancers10100382)

Supplementary Material

Targeting MicroRNA-143 Leads to Inhibition of Glioblastoma Tumor Progression

Eunice L. Lozada-Delgado [1,2,3], Nilmary Grafals-Ruiz [3,4], Miguel A. Miranda-Román [1,3], Yasmarie Santana-Rivera [1,3], Fatma Valiyeva [3], Mónica Rivera-Díaz [2,3], María J. Marcos-Martínez [5,6] and Pablo E. Vivas-Mejía [2,3,*]

[1] Department of Biology, Rio Piedras Campus, University of Puerto Rico, San Juan, PR 00931, USA; eunice.lozada@upr.edu (E.L.L.-D.); mirandar.miguel@gmail.com (M.A.M.-R.); yasmarie.santana@upr.edu (Y.S.-R.)
[2] Department of Biochemistry, Medical Sciences Campus, University of Puerto Rico, San Juan, PR 00936, USA; mrivera@bromediconllc.com
[3] Comprehensive Cancer Center, University of Puerto Rico, San Juan, PR 00935, USA; nilmary.grafals1@upr.edu (N.G.-R.); fvaliyeva@cccupr.org (F.V.)
[4] Department of Physiology, Medical Sciences Campus, University of Puerto Rico, San Juan, PR 00936, USA
[5] Department of Pathology and Laboratory Medicine, Medical Sciences Campus, University of Puerto Rico, San Juan, PR 00936, USA; maria.marcos@upr.edu
[6] Anatomic Pathology Laboratory, Puerto Rico Medical Services Administration, San Juan, PR 00936, USA
* Correspondence: pablo.vivas@upr.edu; Tel.: +1-787-772-8300

Supplementary Material Figure S1. SCL30A8 3'UTR vectors A and B sequences

>HmiT067088a
CTAGCTCAGTCACACCGTCAGTTTCCCAAATTTGACAGGCCACCTTCAAACATGCTGCTATGCAGTTTCTGCA
TCATAGAAAATAAGGAACCAAAGGAAGAAATTCATGTCATGGTGCAATGCACATTTTATCTATTTATTTAGT
TCCATTCACCATGAAGGAAGAGGCACTGAGATCCATCAATCAATTGGATTATATACTGATCAGTAGCTGTGT
TCAATTGCAGGAATGTGTATATAGATTATTCCTGAGTGGAGCCGAAGTAACAGCTGTTTGTAACTATCGGCA
ATACCAAATTCATCTCCCTTCCAATAATGCATCTTGAGAACACATAGGTAAATTTGAACTCAGGAAAGTCTT
ACTAGAAATCAGTGGAAGGGACAAATAGTCACAAAATTTTACCAAAACATTAGAAACAAAAAATAAGGAG
AGCCAAGTCAGGAATAAAAGTGACTCTGTATGCTAACGCCACATTAGAACTTGGTTCTCTCACCAAGCTGTA
ATGTGATTTTTTTTTCTACTCTGAATTGGAAATATGTATGAATATACAGAGAAGTGCTTACAACTAATTTTTAT
TTACTTGTCACATTTTGGCAATAAATCCCTCTTATTTCTAAATTCTAACTTGTTTATTTCAAAACTTTATATAAT
CACTGTTCAAAAGGAAATATTTTCACCTACCAGAGTGCTTAAACACTGGCACCAGCCAAAGAATGTGGTTGT
AGAGACCCAGAAGTCTTCAAGAACAGCCGACAAAAACATTCGAGTTGACCCCACCAAGTTGTTGCCACAGA
TAATTTAGATATTTACCTGCAAGAAGGAATAAAGCAGATGCAACCAATTCATTCAGTCCACGAGCATGATGT
GAGCACTGCTTTGTGCTAGACATTGGGCTTAGCATTGAAACTATAAAGAGGAATCAGACGCAGCAAGTGCTT
CTGTGTTCTGGTAGCAACTCAACACTATCTGTGGAGAGTAAACTGAAGATGTGCAGGCCAACATTCTGGAAA
TCCTATGTCAATGGGTTTGGTTTGGAACCTGGACTTCTGCATTTTTAAAAGTTACCCAGAGATGCTTCTAAAG
ATGAGCCATAGTCTAGAAGATTGTCAACCACAGGAGTTCATTGAGTGGGACAGCTAGACACATACATTGGC
AGCTACAATAGTATCATGAATTGCAATGATGTAGTGGGGTATAAAAGGAAAGCGATGGATATTGCCGGATG
GGCATGGCCAGTGATGTTTCACGTCATTGAGGTGACAGCTCTGCTGGACTTTGAATTACATATGGAGGCTCTC
CAGGAAGACGAAGAAGAGAAGGACATTCTAGGCAAAAAGAAGACTAGGCACAAGGCACACTTATGTTTGT
CTGTTAGCTTTTAGTTGAAAAAGCAAAATACATGATGCAAAGAAACCTCTCCACGCTGTGATTTTTAAAACT
ACATACTTTTTGCAACTTTATGGTTATGAGTATTGTAGAGAACAGGAGATAGGTCTTAGATGATTTTTATGTTG
TTGTCAGACTCTAGCAAGGTACTAGAAACCTAGCAGGCATTAATAATTGTTGAGGCAATGACTCTGAGGCTA
TATCTGGGCCTTGTCATTATTTATCATTTATATTTGTATTTTTTTCTGAAATTTGAGGGCCAAGAAAACATTGA
CTTTGACTGAGGAGGTCACATCTGTGCCATCTCTGCAAATCAATCAGCACCACTGAAATAACTACTTAGCATT
CTGCTGAGCTTTCCCTGCTCAGTAGAGACAAATATACTCATCCCCCACCTCAGTGAGCTTGTTTAGGCAACCA
GGATTAGAGCTGCTCAGGTTCCCAACGTCTCCTGCCACATCGGGTTCTCAAAATGGAAAGAATGGTTTATGC

CAAATCACTTTTCCTGTCTGAAGGACCACTGAATGGTTTTGTTTTTCCATATTTTGCATAGGACGCCCTAAAGA
CTAGGTGACTTGGCAAACACACAAGTGTTAGTATAATTCTTTGCTTCTGCTTCTTTTTGAAAATCATGTTTAGA
TTTGATTTTAAGTCAGAAATTCACTGAATGTCAGGTAATCATTATGGAGGGAGATTTGTGTGTCAACCAAAGT
AATTGTCCCATGGCCCCAGGGTATTTCTGTTGTTTCCCTGAAATTCTGCTTTTTTAGTCAGCTAGATTGAAAAC
TCTGAACAGTAGATGTTTATATGGCAAAATGCAAGAC

>HmiT067088b
GGCCCCAGGGTATTTCTGTTGTTTCCCTGAAATTCTGCTTTTTTAGTCAGCTAGATTGAAAACTCTGAACAGTA
GATGTTTATATGGCAAAATGCAAGACAATCTACAAGGGAGATTTTAAGGATTTTGAGATGAAAAAACAGAT
GCTACTCAGGGGCTTTATGAACCATCCATCAATTCTGAAGTTCTGACTCTCCCATTACCCTTTCCCTGGTGTGG
TCAGAACTCCAGGTCACTGGAAGTTAGTGGAATCATGTAGTTGAATTCTTTACTTCAAGACATTGTATTCTCT
CCAGCTATCAAAACATTAATGATCTTTTATGTCTTTTTTTTGTTATTGTTATACTTTAAGTTCTGGGGTACATGT
GCGGAACATGTAGGTTTGTTACATAGGTATACATGTGCCATGGTGGTTTGCTGCACTCATCAACCTGTCATCT
ACATTCTTTTATGTCTGTCTTTCAAAGCAACACTCTGTTCTTCTGAGTAGTGAAATCAGGTCAACTTTACCACC
AGCCTCCATTTTTAATATGCTTCACCATCATCCAGCACCTACTTAAGATTTATCTAGGGCTCTGTGGTGATGTT
AGGACCCATAAAAGAAATTTATGCCTTCCATATGTTTGGTTACAGATGGGAAATGGGAATGTTGAAGGACAT
GAAAGAAAGGATGTTTACACATTAAGCATCAGTTCTGAAGCTAGATTGTCTGAGTTTGAATCTTAGCTCTTCC
CTTTATTAGCTCTGTGACCTCGAGCTAGTTACTTAAATGCTCTGATCCTCTATTTCCTGATCAGTGAAACCTCC
CTATTCAAATGTGTGAGAGTTTAATAAATTAGGACACTTAAAAATGTTGGAGCAGTGCATAGCATGTAGTGT
TCAGTACATGTTAAATGTTGTTTTTTATTATGTACAAACATGAGTGGGCACAGAATTTTAAATCATCTCAACTT
TTGAGAAATTTTGAGTTATCAACACCGTTCCCACAAGACAGTGGCAAAATTATTGGTGAGAATTAAACAGCT
GTTTCTCAGAGGAAGCAATGGAGGCTTGCTGGGATAAAGGCATTTACTGAGAGGCTGTTACCTAGTGAGAGT
GATGAATTAATTAAAATAGTCGAATCCCTTTCTGACTGTCTCTGAAAGCTTCCGCTTTTATCTTTGAAGAGCA
GAATTGTCACTCCAAGGACATTTATTAATAAAAAGAACAACTGTCCAGTGCAATGAAGGCAAAGTCATAGG
TCTCCCAAGTCTTACCCCATTCCTGTGAAATATCAAGTTCTTGGCTTTTCTCTGTCATGTAGCCTCAACTTTCTC
TGACCGGGTGCATTTCTTTCTCTGGTTTCTAAATTGCCAGTGGCAAATTTGGATCACTTACTTAATATCTGTTA
AATTTTGTGACCCAACAAAGTCTTTTAGCACTGTGGTGTCAAAAAGAAAAACACCTCCCAGGCATATACATT
TTATAGATTCCTGGAGAATGTTGCTCTCCAGCTCCATCCCCACCCAATGAAATATGATCCAGAGAGTCTTGCA
AAGAGACAAGCCTCATTTTCCACAATTAGCTCTAAAGTGCCTCCAGGAAATGATTTTCTCAGCTCATCTCTCT
GTATTCCCTGTTTTGGATCACAGGGCAATCTGTTTAAATGACTAATTACAGAAATCATTAAAGGCACCAAGC
AAATGTCATCTCTGAATACACACATCCCAAGCTTTACAAATCCTGCCTGGCTTGACAGTGATGAGGCCACTT
AACAGTCCAGCGCAGGCGGATGTTAAAAAAAATAAAAAGGTGACCATCTGCGGTTTAGTTTTTAACTTTCT
GATTTCACACTTAACGTCTGTCATTCTGTTACTGGGCACCTGTTTAAATTCTATTTTAAAATGTTAATGTGTGTT
GTTTAAAATAAAATCAAGAAAGAGAGAGTTTGGGTTAAGTCATC

Supplementary Material Figure S2: Sequence alignment of miR-143 and SLC30A8 3′UTR

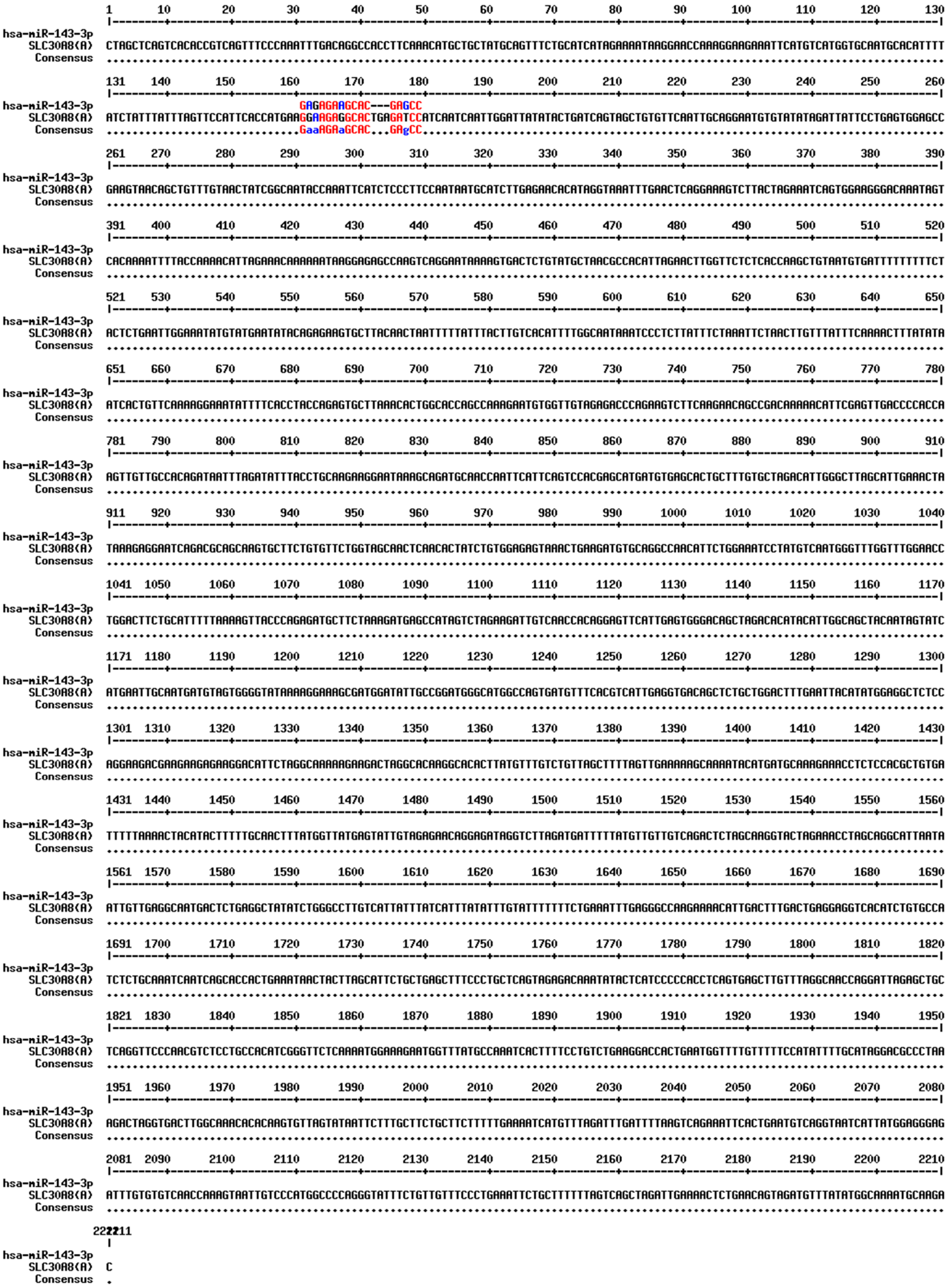

Supplement: Supplementary file 1 [file cancers-10-00382-s001.pdf]
